# A Comprehensive Survey of Small-Molecule Binding Pockets in Proteins

**DOI:** 10.1371/journal.pcbi.1003302

**Published:** 2013-10-24

**Authors:** Mu Gao, Jeffrey Skolnick

**Affiliations:** Center for the Study of Systems Biology, School of Biology, Georgia Institute of Technology, Atlanta, Georgia, United States of America; Princeton University, United States of America

## Abstract

Many biological activities originate from interactions between small-molecule ligands and their protein targets. A detailed structural and physico-chemical characterization of these interactions could significantly deepen our understanding of protein function and facilitate drug design. Here, we present a large-scale study on a non-redundant set of about 20,000 known ligand-binding sites, or pockets, of proteins. We find that the structural space of protein pockets is crowded, likely complete, and may be represented by about 1,000 pocket shapes. Correspondingly, the growth rate of novel pockets deposited in the Protein Data Bank has been decreasing steadily over the recent years. Moreover, many protein pockets are promiscuous and interact with ligands of diverse scaffolds. Conversely, many ligands are promiscuous and interact with structurally different pockets. Through a physico-chemical and structural analysis, we provide insights into understanding both pocket promiscuity and ligand promiscuity. Finally, we discuss the implications of our study for the prediction of protein-ligand interactions based on pocket comparison.

## Author Summary

The life of a living cell relies on many distinct proteins to carry out their functions. Most of these functions are rooted in interactions between the proteins and metabolites, small-molecules essential for life. By targeting specific proteins relevant to a disease, drug molecules may provide a cure. A deep understanding of the nature of interactions between proteins and small-molecules (or ligands) through analyzing their structures may help predict protein function or improve drug design. In this contribution, we present a large-scale analysis of a non-redundant set of over 20,000 experimental protein-ligand complex structures available in the current Protein Data Bank. We seek answers to several fundamental questions: How many representative pockets are there that serve as ligand-binding sites in proteins? To what extent can we infer a similar protein-ligand interaction by matching the structures of protein pockets? How different are the ligands found in the same pocket? For a promiscuous protein pocket, how does a pocket maintain favorable interactions with very different ligands? Conversely, how different are those pockets that interact with the same ligand? We find the structural space of protein pocket is small and that both protein promiscuity and ligand promiscuity are very common in Nature.

## Introduction

At the molecular level, many functions of proteins in a living cell can be attributed to or regulated by their interactions with small-molecule ligands such as metabolites or drugs [1], [2]. A high-resolution structural description of protein-ligand recognition is very important for understanding protein function and designing new compounds for therapeutic purposes. As revealed in many of the crystal structures of proteins in complex with their ligands, protein-ligand interactions usually take place at preferred sites on the protein surface known as “pockets” [3], [4], in contrast to relatively flat geometric shape of protein-protein interaction sites [5]. Traditionally, the study of a protein-ligand complex structures often focuses on the structural or physico-chemical characteristics that are thought to be specific to that individual pocket [6]. However, it is becoming more and more clear that proteins are generally promiscuous in that they interact with multiple distinct ligands [7], [8]. One naturally seeks detailed structural insights into both the origin and generality of this intriguing observation. In this regard, a comprehensive, large-scale comparative study on all protein pockets in all protein structures that are solved to date may uncover principles that explain the promiscuity of protein-ligand interactions.

In such a study, the first question is: How many representative pockets are there in the structural space of all pockets? This echoes a similar question asked about the fold space of proteins [9], [10]. A very recent study addressed this by comparing the pockets of 5,000 single-domain proteins [11]. It was found that a few hundred pocket structures are enough to represent all structure shapes in this set, and similar shaped pockets are also found in artificially generated proteins, which were built and selected based on thermodynamic stability but not biochemical function. In this sense, the structural space of protein pockets is degenerate and surprisingly small. Since the number of known bioactive ligands [12], [13] is much larger than the available number of pocket shapes, the implication is that a given pocket shape can accommodate more than one type of ligand, thus generating the promiscuity responsible for the evolution of biochemical function [14], [15]. The observation that pocket shapes are degenerate suggests that the same ligand could bind to pockets of similar shape but located in different proteins, thus leading to side-effects of drug molecules through unexpected “off-target” interactions [16], [17]. However, the specific interplay of pocket geometry and chemical environment with the types of ligands that are bound was not addressed in that study [11] as it focused on the properties of pockets in proteins without a companion analysis of the bound ligands. In the current contribution, we address this issue.

A second question is: To what extent can we infer a similar protein-ligand interaction by matching protein pockets? The answer to this question has practical applications for protein function prediction [18] or small-molecule compound screening [19]. In order to match pockets, many computational approaches have been developed to compare pockets based on their structural and/or physico-chemical features (for a review see [20]). These methods may be categorized into two classes: The first is based on the structural alignment of pocket-lining residues or atoms [21]–[24], and the second is based on comparison of descriptors independent of the residue or atom alignment [25]–[27]. The former class is generally more accurate, albeit slower than an alignment-free method, due to the complexity of the alignment algorithm. In that regard, we recently proposed an efficient, robust method, APoc, for large-scale pocket comparison [28]. On the other hand, since a structural alignment is not required, alignment-free methods might have an advantage in dealing with flexible pockets. Their disadvantage is that they often lack a direct physical interpretation for why two pockets are similar as assessed by their fingerprints.

Another interesting question is: How different are the ligands that bind to the same pocket? Obviously, if the ligands are very similar, they are very likely to have similar interactions with the pocket, e.g., that might contain a common anchor and variable region [29]. However, if the ligands possess different scaffolds and/or chemical properties, it might not be obvious as to what, if any, interactions are conserved. How does a pocket maintain favorable interactions with very different ligands? Conversely, a ligand may be found in pockets of different protein structures. How different are those pockets that interact with the same ligand? An early study of pockets from non-homologous proteins that bind the nine most common ligands suggests that there are shape variations in these pockets [30]. This further raises the question of how a ligand manages to interact with different pocket shapes.

To address these questions, we performed a comprehensive comparative study on a large curated set of over 20,000 ligand-bound pocket structures from crystallized protein-ligand complexes. We first characterize the structural space of these pockets. This is followed by an analysis of the correlations between pocket similarity and ligand chemical similarity. Then, we investigate both *pocket promiscuity* (one pocket accommodating different ligands separately) and *ligand promiscuity* (one ligand recognized by different proteins), respectively. Finally, the implications of our study are discussed.

## Results

### How many representative protein pockets involving ligand-protein recognition are there?

**Figure 1:**
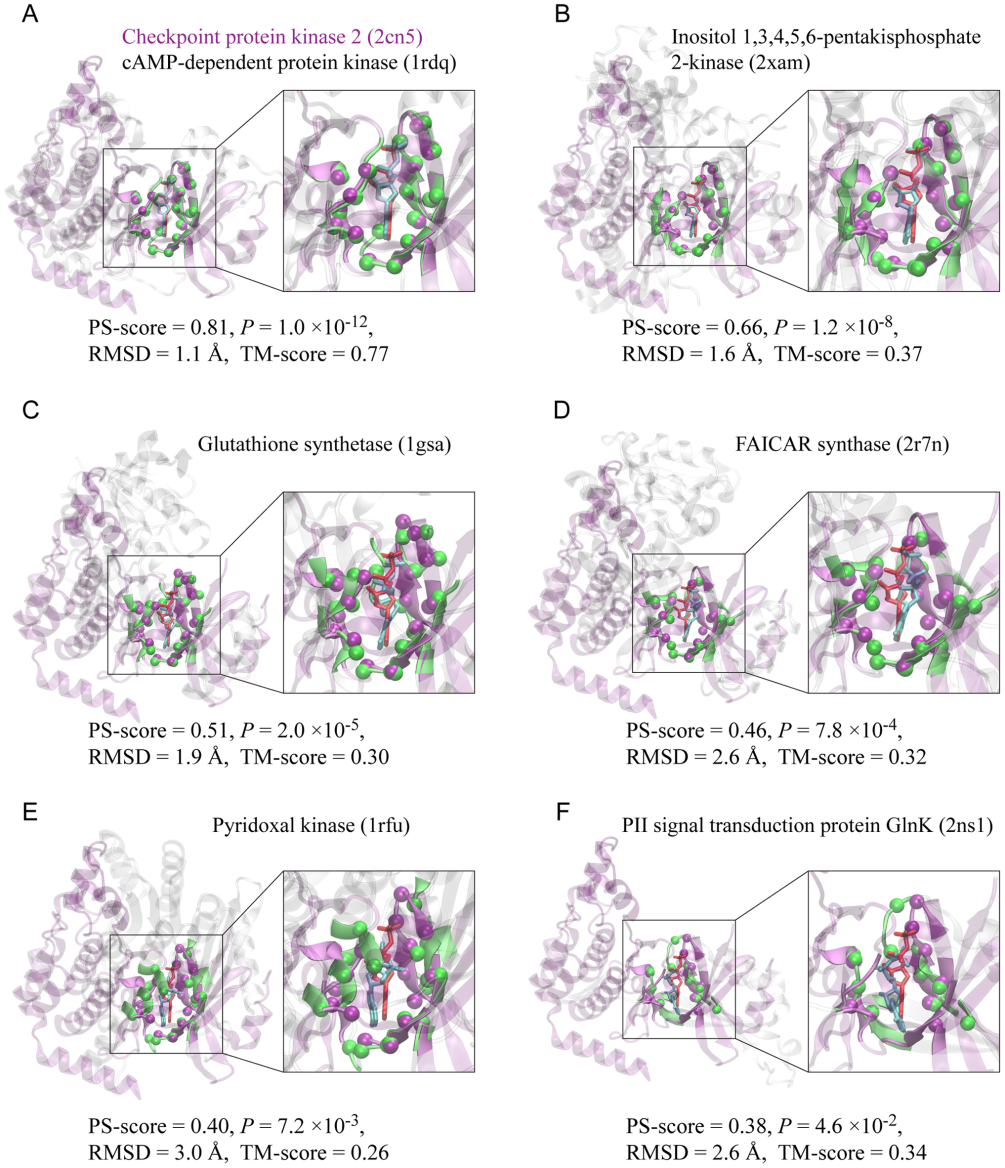
Examples of pocket alignments according to APoc. (**A–F**) Six ADP-binding pockets taken from six different protein structures (green) are aligned to a common ADP-binding pocket from the checkpoint protein kinase Chk2 (purple). In each snapshot, the two protein structures are shown in cartoon representations, and the corresponding bound-ligands are shown in cyan and red licorice representations, respectively. For clarity, non-pocket regions are shown in transparent purple in Chk2, and in transparent grey in the other proteins, whereas pocket regions are shown in solid purple in Chk2 and solid green in the other cases. Aligned pocket Cα atoms are shown as spheres. An enlarged view of the pocket alignment is displayed on the right. The top label denotes the name of the protein and its PDB accession code in parentheses; and the bottom label denotes the corresponding PS-score, *P*-value, RMSD of aligned atoms, and the TM-score. Molecular images were created with VMD [56]. They were taken in the same view at Chk2.

To answer this question, we have collected all crystal structures of protein-ligand complexes deposited in the PDB till May 2012 and curated a non-redundant set of 20,414 ligand-bound pockets, which contains 9,485 unique ligands (see Methods). A pocket is defined by ligand-binding sites, i.e., the amino acids in physical contact with the ligand. We then performed all-against-all pocket comparisons using the pocket comparison method APoc [28]. Pocket similarity is evaluated by the pocket similarity score (PS-score), which measures the geometry of backbone Cα atoms of aligned pocket-lining residues, as well as their side chain orientation and chemical properties. Identical pocket structures have a perfect PS-score of 1. Significant similarity emerges starting from a PS-score higher than 0.36 (see Table S1). Fig. 1 shows the APoc alignments of six adenosine diphosphate (ADP) binding pockets from six different proteins against a common ADP-binding pocket from protein kinase Chk2 [31]. These examples illustrate pocket similarity at various significance levels of their PS-scores. In the first example (Fig. 1A), another protein kinase [32], a homolog of Chk2, matches Chk2 both the pocket and global fold structures at a PS-score of 0.81, an associated *P*-value of 1.0×10^−12^, and a Template Modeling score (TM-score) of 0.77. TM-score is a measure for protein global structural similarity, and a TM-score higher than 0.40 is significant [33]. In the other five cases, there is low or no global structural similarity, reflected by both visual inspection and low TM-scores of no more than 0.37. However, APoc detects similarity in their ligand-binding pockets. An inositol phosphate kinase [34] exhibits a strong resemblance to Chk2 in their pockets at a PS-score of 0.66, a *P*-value of 1.2×10^−8^, and an RMSD of 1.6 Å in the aligned pocket-lining Cα atoms (Fig. 1B). Two proteins in ATP-grasp folds, a glutathione synthetase [35] and a FAICAR synthase [36], display highly significant similarity at PS-scores of 0.51 and 0.46, together with *P*-values of 2.0×10^−5^ and 7.8×10^−4^, respectively. The last two examples, a pyridoxal kinase [37] and a signaling protein GlnK [38], show lower pocket similarity to that of Chk2 at PS-scores of 0.40 and 0.38, and *P*-values of 7.2×10^−3^ and 4.6×10^−2^, respectively. In these two cases, there are some adjustments by ligands in their docking poses in response to the structural variations of their pockets, yielding relatively low, but still significant PS-scores.

We then seek to find the smallest set of pockets (or templates) that are sufficient to represent the full set of pockets at a given level of similarity. In terms of graph theory, pocket similarity relationships can be viewed as a directed graph *G*, wherein each node defines a pocket, and an edge from pocket A to pocket B indicates that A as a representative pocket has significant similarity to B above a specified PS-score threshold. Thus, the sought-after set of representative pockets is the smallest dominating set of the graph *G* (see Methods).

**Figure 2:**
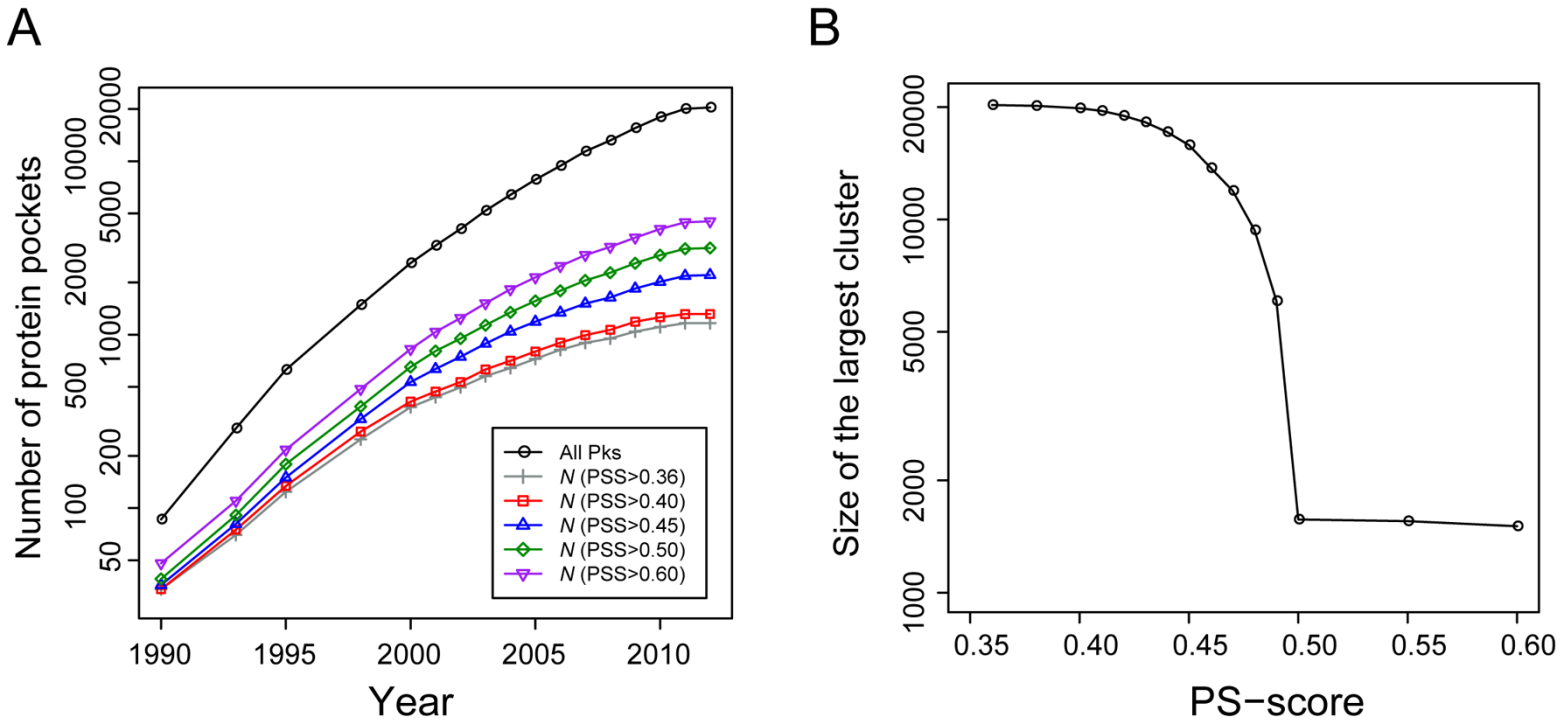
Representative protein pockets for ligand-binding in the PDB. (**A**) Number of representative pockets versus year. “All Pks” denotes all 20,414 non-redundant pockets collected from the PDB up to May, 2012. The number of representative pockets was obtained by finding the smallest dominating set of all pockets at a specified PS-score (PSS) and a significant *P*<0.01. The number of pockets is shown on a logarithmic scale. (**B**) Size of the largest cluster of pockets at different PS-scores. Each PS-score threshold defines a graph representing the structural relationships of pockets. In each graph, the largest cluster forming the LSCC is then identified, and the size of the LSCC is plotted against the PS-score threshold.

Fig. 2A shows the growth of representative protein pockets versus year. As background, the total number of pockets examined exhibits an exponential growth, especially from the years 1990 to 2000. After this initial rapid increase, however, the annual growth rate has been gradually slowed down from 26% in 2001 to 15% in 2011. The trend is similar in *N*, the number of selected representative pockets, but the pace of growth is even slower. For example, at a PS-score threshold of 0.40, the annual growth of *N* decreased from 14% in 2001 to 4% in 2011; at the PS-score of 0.50, the rate is 24% in 2001 and 9% in 2011. These results suggest that many pockets are structurally redundant, e.g., the highly similar ATP-binding pockets from a large family of protein kinase catalytic domains that happen to be the binding-sites of many designed inhibitors as well.

The observation that the number of representative pockets is approaching a plateau at a significant PS-score of 0.40 supports the notion that the structural space of ligand-bound pockets is close to complete, and a set of 1,315 pockets may represent the current pocket library at this similarity level. Pairwise comparisons between matched target pockets and these representatives give a mean alignment RMSD of 1.74 Å, a mean alignment coverage of 84%; half of these comparisons have a highly significant *P*<1×10^−4^ (Fig. S1). Note that this number of representative pockets is higher than that reported in a previous study [11], which found 339 representatives in 5,000 proteins of less than 250 residues. If we use the same protein length criterion, the total number of pockets is reduced 65%, and a total of 332 representative pockets were obtained at a PS-score of 0.40. These numbers are therefore consistent. At a high PS-score of 0.50, a set of 3,158 representative pockets are selected, and about 96% of matching pocket comparisons have a RMSD of 2.5 Å or less, 90% have an alignment coverage better than 70%, and 94% with a *P*<1×10^−4^.

From a network prospective, the structural space of pockets is highly connected, meaning that virtually all pocket nodes can reach other pocket nodes through a path of significantly related pockets; that is, the Largest Strongly Connected Component (LSCC) dominates *G*. About 97% of all pockets belong to the LSCC at a PS-score of 0.40, and the percentage is 75% at 0.45 (Fig. 2B). Notably, a phase transition occurs at a PS-score threshold of 0.50, when the space becomes disconnected with 1,834 strongly connected components (or clusters), and the corresponding LSCC consists of only 7.7% of all pockets. At this level, the pocket space becomes discrete and members in the same cluster could be evolutionarily related. For instance, the LSCC at PS-score of 0.50 is composed of 1,571 ATP- and ADP-binding pockets, about 90% of them are from protein kinases, and the remaining from likely related proteins whose function is also dependent on ATP, such as glutathione synthases, SAICAR synthases, and some other types of kinases. Some examples are shown in Fig. 1.

### Can one infer ligand-binding based on pocket similarity?

**Figure 3:**
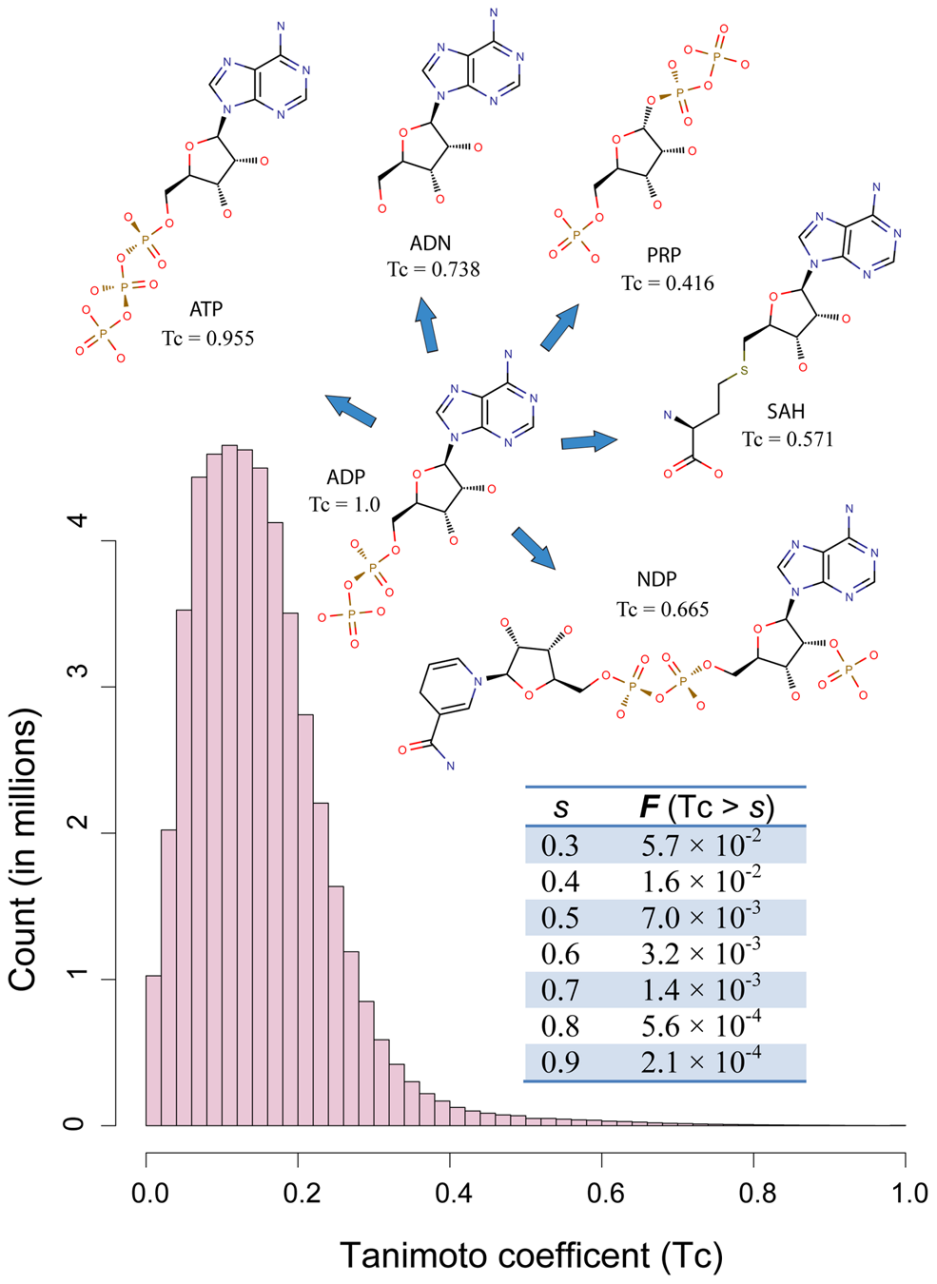
Distribution of Tanimoto coefficient scores of small-molecule compounds found in the PDB. Tc scores from all-against-all comparison of 9,485 ligands were employed to create the histogram. Insert table shows the fraction of Tc scores higher than threshold scores. Insert diagrams display chemical structures of ADP and five structurally related ligands: adenosine triphosphate (ATP), adenosine (ADN), NADPH dihydro-nicotinamide-adenine-dinucleotide phosphate (NDP), S-adenosyl-L-homocysteine (SAH), and α-phosphoribosylpyrophosphoric acid (PRP). Their Tc scores in comparison to ADP are provided under their name labels.

A common assumption for inferring protein-ligand interaction is that similar pockets bind similar ligands. The relationship between ligand similarity and pocket similarity, however, needs a thorough examination. Here, we use a 1024-bit fingerprint to compare the chemical similarity of ligands in terms of their pairwise Tanimoto coefficient (Tc, see Methods). As shown in Fig. 3, the distribution of all-against-all (excluding self comparison) Tc values of 9,485 ligands in our data gives a mean Tc value of 0.162 and a standard deviation of 0.088. The distribution has a long tail, suggesting that there exist many similar ligands in our set. A Tc score higher than 0.4 appears in less than 2% of all cases. In our analysis below, Tc scores above 0.4 are deemed significant. Five ligands whose structures are related to ADP are demonstrated as examples in Fig. 3. These ligands have Tc values ranging from 0.4 to above 0.9.

**Figure 4:**
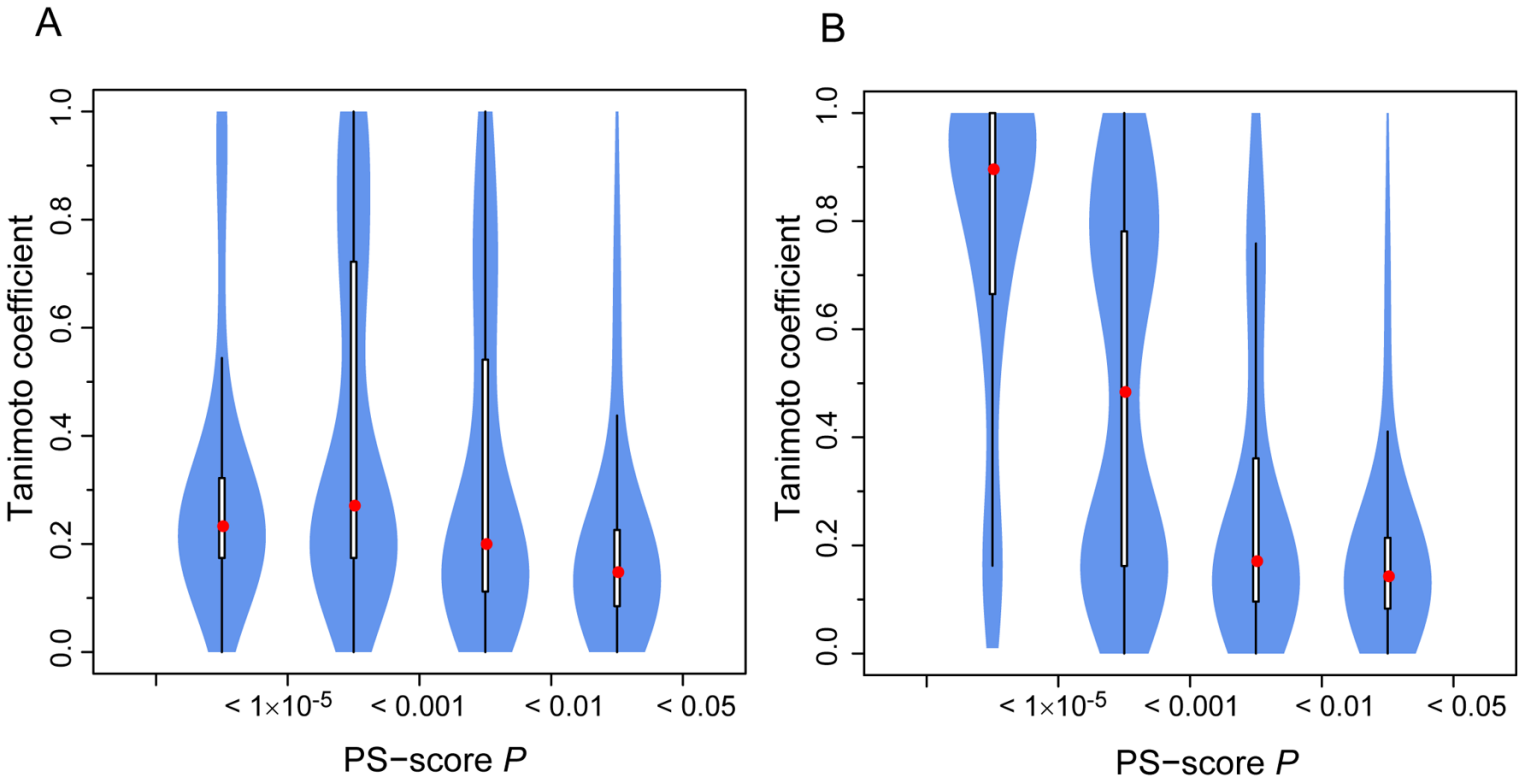
Violin plot of chemical similarity of ligands found in structurally similar pockets. (**A**) All 7 million pairs of pockets at PS-score *P-values*<0.05 are considered. The *x*-axis labels mark similarity regimes for pocket pairs considered. (**B**) The subset of pocket pairs from proteins with low pairwise global structural similarity at the TM-score <0.4. A Violin plot is derived from a boxplot by scaling the width of the box such that the area is proportional to the number of pairs of ligands observed. The white bars range from 25^th^ to 75^th^ percentile, and the whiskers extend to a distance of up to 1.5 times the interquartile range. The red spheres represent the medians.

Fig. 4A shows the distribution of ligands at different pocket similarity levels, defined by the *P-values* of their PS-scores. For a 0.01≤*P-value*<0.05, about 13% of ligand pairs share significant chemical similarity at a Tanimoto coefficient (Tc)>0.4. This percentage increases to 31% and 37% as one increases the pocket similarity level to a *P-value* of 1×10^−3^ and 1×10^−5^, respectively. The percentage drops to 18% at pocket *P*<1×10^−5^. This unexpected observation is due to many pockets are promiscuous and interact with chemically different ligands. The PDB is biased towards these types of pockets, because they are often from putative drug targets, e.g., protein kinases, proteases, etc.

In some cases, it is possible to identify local pocket similarity when overall global structural similarity is likely absent. Fig. 4B displays only those cases where pockets are from proteins with different global structures at a TM-score <0.4 [33]. About 24% and 52% of pockets recognize similar ligands at a Tc>0.4, at a corresponding *P-values* of 1×10^−3^ and 1×10^−5^, respectively. The percentage increases to 84% for highly similar pockets at *P*<1×10^−5^. However, we note that the number of cases considered here is much smaller than Fig. 2A and might therefore underestimate pocket promiscuity. The regime where *P*<1×10^−5^ comprises of only 0.7% of pockets. The subset is dominated by GDP-binding pockets that appear in multi-domain proteins with low global similarity but high pocket similarity. Nevertheless, the analysis shows that it is possible to detect pockets that share both similar ligands and pockets, even though they may be from two proteins with very different global structures.

**Figure 5:**
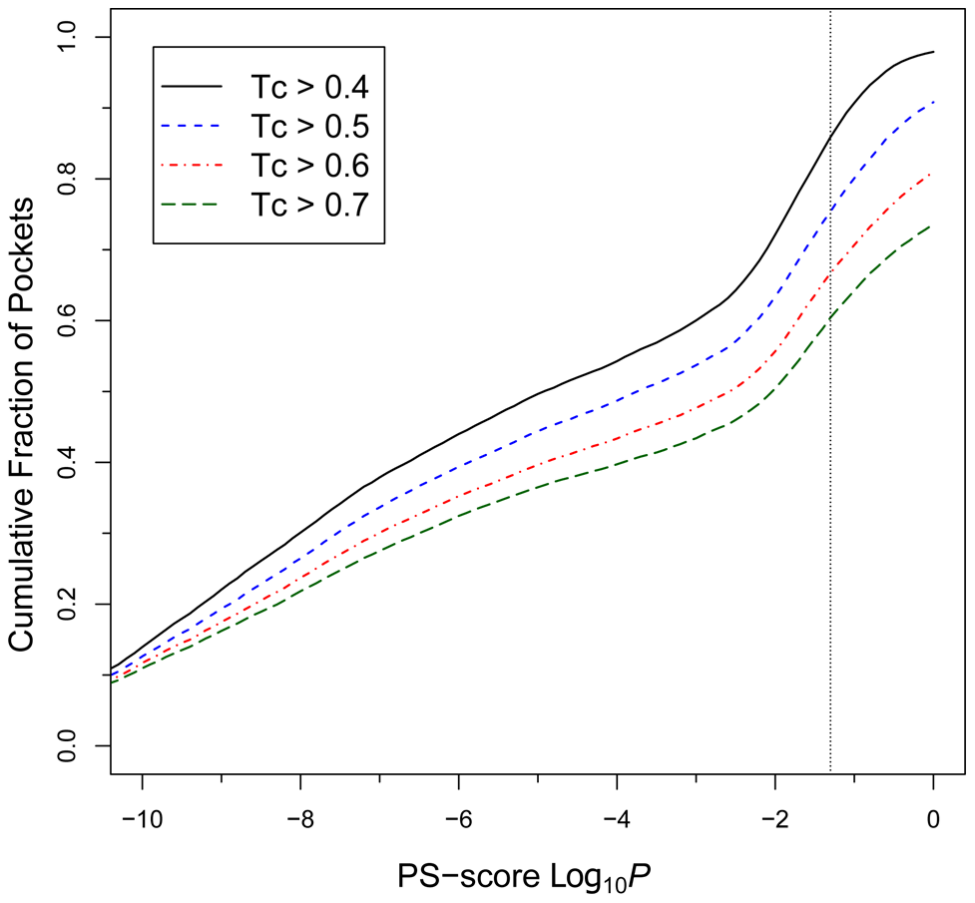
Cumulative fraction of 20,414 pockets matched by templates at a similarity level better than the given PS-score *P-value*. Curves are generated separately at different levels of ligand similarity as measured by Tc. The vertical dotted line is located at a *P-value* = 0.05 for the PS-score.

Next, we ask the question of how many protein-ligand interactions observed in the PDB can be matched to a template that has both a similar pocket and a similar ligand? To answer this question, for each target pocket, we search for the best structural hit that satisfies two conditions: (i) global sequence identity <30%; and (ii) chemical similarity Tc of ligands larger than a specified value. The result is shown in Fig. 5. At a significant Tc>0.4, about 86% of pockets can find a template hit with significant pocket similarity at the PS-score *P*<0.05 that binds similar ligands. The numbers are 72%, 60%, and 54% at *P*<0.01, 0.001, and 0.0001, respectively. At a highly significant Tc>0.7, most (60% and 50% of) pockets hit a template at *P*<0.05 and <0.01, respectively. The result shows that structural comparison of pockets could be useful for inferring ligand-binding. In particular, many of these top structural hits come from proteins with low global structural similarity or even different structural folds. At a Tc>0.4 and *P*<0.05, about 35% of the top template hits are from proteins with global TM-score <0.4. The percentage is 19% at a Tc>0.7. These are challenging cases for sequence-based methods, but could in principle be dealt with by adopting a structure-based approach. However, we also note that for all Tc values, there remain a significant fraction of pockets that are structurally unrelated and yet they bind similar ligands.

### Many protein pockets are promiscuous

The above results indicate that pockets of similar shapes can attract a diverse set of ligands with different chemical properties. One obvious explanation that accounts for this observed chemical diversity is that a similarly shaped pocket may have a different amino acid composition, thus generating different physico-chemical environments favored by chemically different ligands, e.g., homologs with modified substrate specificities. A second reason is that, for large pockets, some small-molecule ligands may be bound to at least partially different regions of the pockets, and these ligands may not necessarily have similar chemical properties.

**Figure 6:**
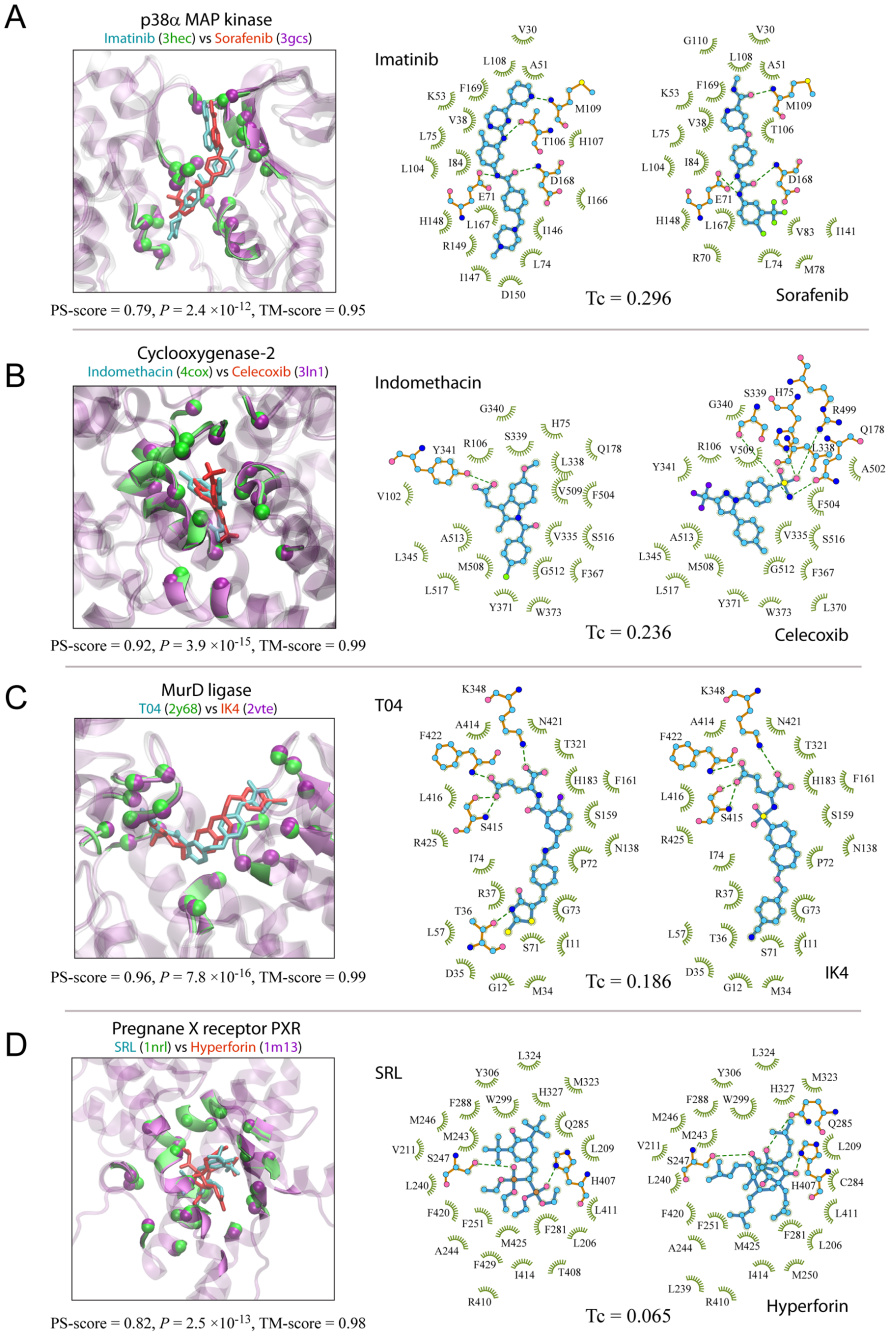
Four examples of promiscuous pockets recognized by ligands of different chemical structures. (**A–D**) Each panel is composed of three snapshots. On the left is the APoc superimposition of the same protein pockets separately in complex with two ligands. The representation is the same as in Fig. 1. Labels of ligands and PDB codes (in parentheses) are in the same color scheme as their 3D images. On the middle and right are the schematic 2D views of the two ligands and their respective interacting pocket residues. Ligands are shown in a stick and ball representation. Protein residues that form hydrogen bonds are also shown in a stick and ball representation, and other contacting residues are shown in a green eyelash representation. In the stick and ball representation, carbon, oxygen, nitrogen, phosphorus, sulfur, chlorine, fluorine atoms are shown as cyan, red, blue, brown, yellow, green, purple balls, and covalent bonds in the ligand and protein are shown in cyan and orange sticks. Hydrogen bonds are indicated by green dashed lines, with their lengths (all less than 3.35 Å) not drawn to scale. Amino acids are labeled by their one-letter code followed by their residue index in the original PDB records, except for 4cox in (B), whose residue indexes are renamed to be consistent to 3ln1 for clarity. Diagrams of ligand-protein interactions were created with the program LigPlot+ [57].

Of special interest are promiscuous pockets, i.e., the same pockets recognized by ligands with different chemical structures. To examine pocket promiscuity, we selected a set of 59,157 pairs of pockets of comparable size, each pair having a highly significant PS-score >0.6, sequence identity = 100%, and bound ligands at low Tc<0.3. These pockets are essentially from the same proteins crystallized with different ligands. The set is composed of 6,913 unique pockets, or 34% of all pockets in our set, and they are from 421 different clusters determined at a PS-score of 0.50. At this level, about 25%, 31%, and 36% of all pocket clusters with more than 2, 10, and 20 members contain at least one promiscuous pocket, respectively. Thus, it is clear that promiscuous pockets are not rare at all. Fig. 6 shows four examples. In each case, the same pocket is shown to interact with two ligands of different structures. Perhaps, the most well-known examples are ATP-binding pockets of protein kinases, for which many novel inhibitors have been designed. Two such examples are shown in Fig. 6A, where a protein kinase p38α accommodates two drugs, Imatinib [39] and Sorafenib [40], thereby inhibiting ATP from binding at the same pocket. Although these two inhibitors were originally designed to target different protein kinases and cancer types, they have been shown to interact with other protein kinases such as MAP kinase p38α. In the second example (Fig. 6B), two anti-inflammatory drugs, Indomethacin [41] and Celecoxib [42], are demonstrated to interact with a common protein target, cyclooxygenase-2 (COX-2). Both drug molecules bind to the active site of the enzyme. The third example involves MurD ligase, which catalyzes the formation of peptidoglycan ubiquitously in bacteria but is absent in human; thus, it is an attractive target for the design of novel anti-bacterials. Fig. 6C depicts two experimental compounds intended for this target. They are both N-substituted derivatives of d-Glutamic acids, and are recognized by the same set of active site residues of the enzyme [43], [44]. Last, we present a well-known promiscuous protein, pregnane X captor (PXR), which is a nuclear receptor protein responding to a variety of endogenous and exogenous chemicals. Fig. 6D displays the interaction of PXR with two compounds [45], [46], which have a very different chemical structure at a very low Tc of 0.065, yet they are found in the same, largely hydrophobic pocket.

**Figure 7:**
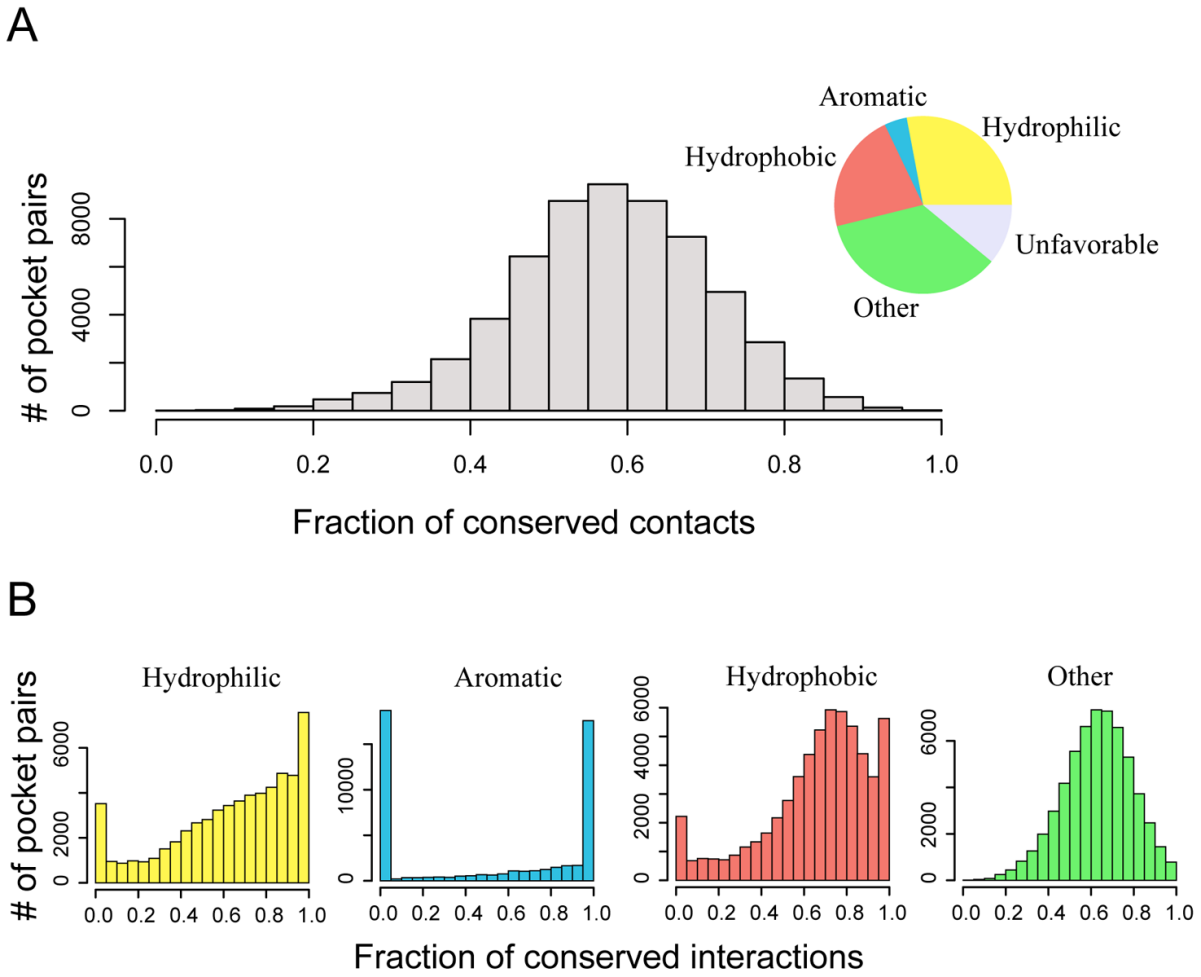
Distribution of conserved contacts between dissimilar ligands (Tc<0.3) bound to the same pockets. (**A**) Overall distribution of all conserved contacts that are not unfavorable. The inserted pie chart shows ligand-protein interactions by type according their contributions to the overall contact surface area. (**B**) Distributions of individual types of ligand-protein interactions that are conserved between two pairs of ligand/pocket interactions.

How does a promiscuous pocket carry on interactions with different ligands? We decomposed atomic contacts at the protein-ligand interfaces of the above 59,157 complex structures. On average, 28%, 22%, and 4% of interactions are hydrophilic, hydrophobic, or aromatic, respectively; the remaining are either neutral (or slightly favorable interactions, 35%) or energetic unfavorable (11%) interactions. As shown in Fig. 7, a comparative analysis revealed that most (58%) physical interactions of similar type are conserved between pairs of complexes. Individually, 64% of hydrophilic or hydrogen-bond interactions, 53% of aromatic interactions, and 66% of hydrophobic interactions are conserved on average. Since aromatic contacts are rare in some structures, they are not required to be conserved to maintain stable protein-ligand interactions, yielding the peak at zero conserved interactions observed in Fig. 4B. Overall, even though ligands may have a very different scaffold, they may achieve the same physical interactions with the same pocket residues. Second, specific contacts (i.e., hydrophilic or hydrogen-bonding interactions) contribute only 28% of all contacts on average. As a result, favorable interactions are more flexible than might be expected on average. Finally, the plasticity of protein pockets may allow different types of interactions [11]. The mean PS-score of these pockets is 0.86, and most cases have a *P-value*<1×10^−12^. These are highly similar but clearly not identical pockets. The flexibility of side chains permits different types of contacts with different ligands to form.

### The same ligand is often recognized by different pockets

**Figure 8:**
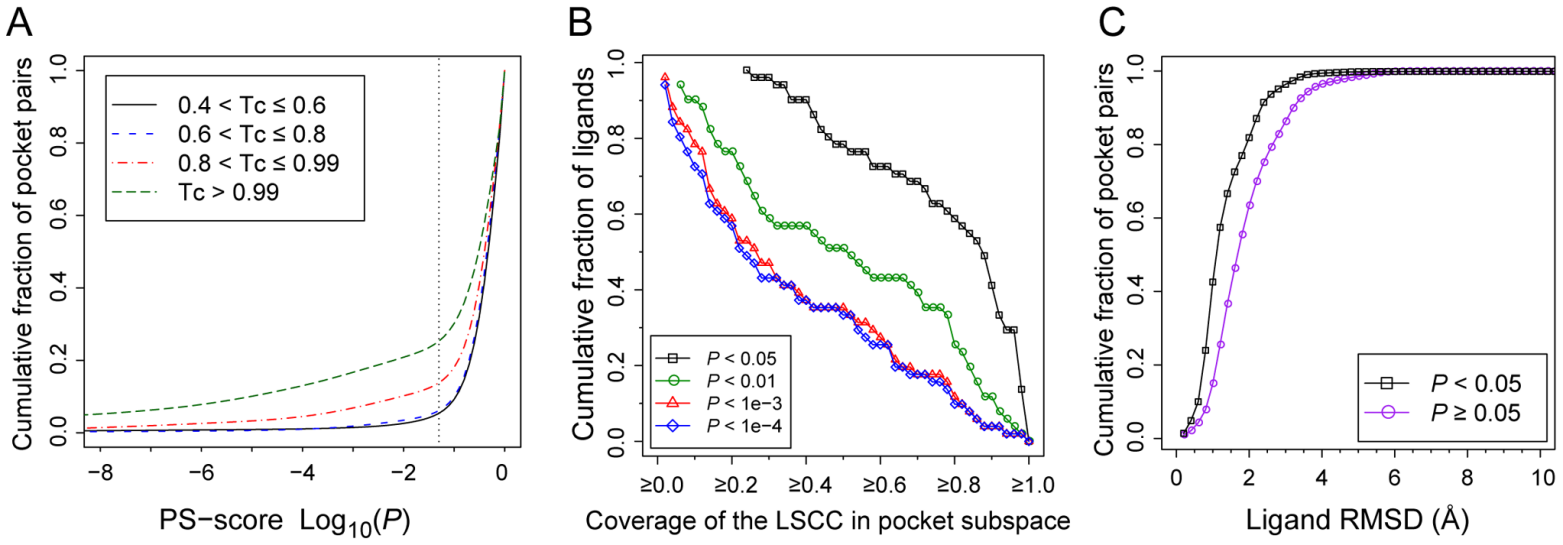
Statistics of protein pockets recognizing similar or identical ligands. (**A**) Cumulative fraction of pocket pairs at a pocket similarity better than then given PS-score *P*-value. Each pair of pockets bind to similar or identical ligands in various Tc regimes. The dotted line is located at *P* = 0.05. (**B**) Cumulative fraction of ligands versus the coverage of their largest pocket cluster defined by the LSCC. The coverage is the size of the LSCC divided by the number of all pockets within each ligand's pocket space. (**C**) Cumulative faction of identical ligand pairs with an atomic RMSD less than a given value. Ligand pairs are categorized into two groups according to the similarity of their corresponding pockets. A PS-score *P* of 0.05 was employed as the threshold for the categorization.

Finally, we perform an analysis on pockets that accommodate chemically similar or identical ligands. Fig. 8A shows the structural similarity of pockets that recognize similar ligands at various but significant Tc values >0.4. For Tc≤0.8, it is clear that most pocket pairs are structurally dissimilar, with only about 5–6% of pocket pairs having a significant PS-score, even though they recognize similar (but not identical) ligands. The fraction of similar pockets pairs at *P*<0.05 increases to 14% for 0.8<Tc≤0.99. Thus, even here, on average very similar ligands interact with structurally distinct pockets. Furthermore, 66% of pockets in our set interact with virtually the same ligand (Tc>0.99) that binds to at least one other pocket. This set includes 1,475 unique ligands, and about 25% and 13% of pocket pairs binding the same ligand share a similarity at *P*<0.05 and 0.0001, respectively. Thus, many ligands are promiscuous and interact with structurally different pockets.

We further focus on a set of 5,991 pockets bound to 51 of the most frequently observed ligands (see Table S2), each with more than 30 distinct pockets. For each ligand, we gathered all its pockets, which forms pocket subspaces of ligands, converted pocket similarity relationship into graphs, and subsequently performed graph analyses. As shown in Fig. 8B, for 78% of these ligands, more than half of their pockets are clustered together to form the LSCC at a *P-value*<0.05 in their respective subspaces. The percentage is 51% at a *P-value*<0.01. This result implies that at least a fraction of structural features are conserved between some of these pockets within the LSCC cluster, though the substructure conservation is not necessarily always transitive. Thus, most pockets are structurally related, albeit some at low level of similarity. Nevertheless, for each type of ligand, one may represent the entire relevant pocket space using a few representative pockets, dependent on desired level of similarity, as shown in Table S2. For example, one needs 31, 19, 23, and 15 pockets to cover 456, 431, 371, and 329 observed pockets at *P*<0.05 for ADP, HEM, NAD, FAD, the top four mostly common ligands in the set, respectively.

The result that the same ligand may interact with different pockets suggests that there exists multiple interaction poses between the ligand and its pockets. One major contributing factor to the multiple interacting poses is the conformational change of the ligands. Fig. 8C shows the cumulative distribution of the atomic RMSD for the same ligands observed in the similar pockets (*P*<0.05) versus the dissimilar pockets (*P*≥0.05). In about 70% and 82% of similar pockets, the corresponding ligand RMSD is less than 1.5 and 2.0 Å, respectively, versus 42% and 63% of the dissimilar pockets. In addition, the same conformer of a ligand may interact with different pockets in different poses [28]. They are the most challenging cases for a structure-based prediction on ligand-protein interactions.

## Discussion

Our study demonstrates a complicated picture of protein-ligand interactions. First, from mainly a structural prospective, the space of the protein pockets is degenerate. The growth rate of novel pockets deposited in the PDB has been steadily decreasing over the past decade, approaching a plateau. At a PS-score of 0.40 (*P*<0.01), one can find a structural match for all known pockets by using about 1,300 representative pockets. The number is higher than that reported in an earlier study [11], which was limited to proteins with less than 250 residues and employed a less stringent pocket similarity criterion. Perhaps, this result is not that surprising given that the structural space of protein folds themselves is also finite [9], [10]. Like protein fold space, the structural space of protein pockets is continuous in the sense that a significant set of structural features in one pocket can be found in another pocket, which may not share any evolutionary relationship. Interestingly, at a high structural similarity level (PS-score 0.50), a phase transition occurs in pocket space (Fig. 2B), yielding mostly isolated clusters of pockets that could share an evolutionary relationship. However, this is not to say pockets at a lower similarity level do not share an evolutionary origin or that those at a higher similarity level have a common ancestor. Instead, it means that it is difficult to establish their evolutionary relationship using structural information alone. This observation is analogous to a “continuous-to-discrete” view of protein fold space [47].

Like the classification of protein folds, classification of protein pockets is dependent on the similarity criteria employed. We note that there is no perfect metric or criteria that gives a universally agreed upon classification. In our study, pocket similarity comparison focused on the position of Cα and Cβ atoms of the pocket-lining residues, as well as their chemical similarity. The similarity criteria we selected are based on estimation of the statistical significance, ranging from *P*<0.05 to highly significant *P*<1×10^−6^ according to APoc [28].

One major purpose of pocket comparison is to develop a structure-based method for predicting protein-ligand interactions. The rationale behind is that similar pockets attract similar ligands. This is certainly true to some extent; as shown in Fig. 5, for 72% and 50% of ligand-bound pockets one can find another similarly shaped pocket (at *P*<0.01) that interacts with a similar ligand at a Tc>0.4 and 0.7, respectively. One advantage based on structural comparison of pockets is that one may uncover ligand-protein interactions that are undetectable from sequence or global structural comparison. However, there is one limitation to this approach. As we shown here, one type of pocket shape can accommodate multiple types of ligands, which could introduce false positives. To address this issue, it is necessary to increase the level of pocket similarity to reduce false positives, at the cost of sensitivity. This explains why current methods have relatively low coverage in benchmark tests [23], [28]. How to improve sensitivity and maintain a low false positive rate remains a challenge for predicting protein-ligand interactions on the basis of pocket similarity.

Many protein pockets are promiscuous. More than 1/3 of pockets in our data set belong to those promiscuous pockets that interact with multiple, chemically different ligands. Considering that only a tiny fraction of protein-ligand interactions are captured in the PDB, the results shown here likely represent a lower bound, and it is very likely that promiscuous protein pockets are more common. From an analysis of protein-ligand interactions observed in promiscuous protein pockets, we showed that a large fraction (∼60% on average) of these interactions share similar types of interactions, e.g., hydrogen bonding, hydrophobic, or aromatic. Moreover, the plasticity of protein pockets may also provide alternative, viable interaction modes [11]. Therefore, these promiscuous interactions may be understood from a physical chemical point of view. In principle, if one could design a scheme that matches similar ligands based on their physico-chemical properties regardless of their chemical scaffolds, then it could provide a means of predicting novel protein-ligand interactions. In practice, however, this is a highly challenging problem because many physical interactions such as hydrophobic interactions are not very specific, thus allowing many possible solutions that increase the chance of hitting a false positive.

The complexity of protein-ligand interactions is also reflected in ligand promiscuity. That is, a ligand with different poses may interact with differently shaped pockets. One main reason is that ligands with multiple rotatable bonds are flexible, thus yielding different conformations selected by different pockets. In some cases, different poses fit different physico-chemical environments [48]. These observations further help explain polypharmacology or the unexpected “off-target” interactions found in many drug molecules [17]. From a prediction point of view, for a compound of interest, it is unlikely to predict all its protein partners based on only one template because of ligand structural diversity. In this regard, a catalog of structures of many-faceted protein-ligand interactions could significantly improve the prediction of side-effects or repurposing of drugs.

In summary, we find that both protein pocket promiscuity and ligand promiscuity are common. The relationship of protein pockets and ligands is often not one to one but many to many. A given ligand may interact with a number of proteins whose structures are globally unrelated but contain similar pockets. Or it might interact with proteins having different pockets. Conversely, a given pocket can have similar physico-chemical interactions with ligands that may or may not have similar linear structures. For the case of dissimilar ligand pairs, they can adopt conformations that have similar interaction surfaces. Based on this and prior work [7], we conclude that promiscuous ligand interactions of differing specificity are inherent to proteins and living cells. This has a number of implications: It provides a mechanism for a living cell to select for useful biochemical functions as such low level function is likely inherent to a soup of quasi stable protein structures which can then be optimized [14], [15]. It also provides biological robustness [49]. On the other hand, it could cause difficulty in the control of biological processes and in assessing the accuracy of predicted protein-ligand interactions, since we are far from knowing all protein-ligand interactions. This work clearly argues that the notion of one ligand-one protein target that implicitly underlies many drug discovery efforts is fundamentally incorrect.

## Methods

### Data set

We collected a set of 20,414 ligand-bound pockets from holo-protein structures in the PDB [6]. The data set is curated from all 81,756 entries in a May 2012 PDB release. The program LPC [50] was applied to analyze protein-ligand complex structures. For each protein-ligand complex, the program returns a table of protein residues contacting with the ligand. A protein-ligand contact is defined based on the distance between heavy atoms from the protein and from the ligand, respectively. If the distance of a pair of atoms is less than the sum of the Van der Waals radii of the two atoms plus 2.8 Å, which is the diameter of a probing solvent molecule, then the residue that the protein atom belongs to is considered a pocket residue. All such residues collectively compose a protein pocket. In this study, we consider small molecule ligands that have at least ten and fewer than 200 heavy atoms, but do not consider polypeptides, DNA, or RNA molecules. In the PDB, each type of ligand is represented by a unique three-letter name known as the HET code. If one PDB entry contains multiple ligands with an identical HET code, we arbitrarily select the ligand making the most contacts with the protein. The primary protein chain that a ligand associates with is clustered at 90% sequence identity. In each cluster, we subsequently select a representative for each type of ligand, using X-ray structure resolution and number of contacts as the selection criteria. Finally, we discarded pockets with l0 or fewer residues. This yields 20,414 ligand-bound pockets, which are bound to 9,485 unique ligands.

The chemical similarity of ligands is measured by their pairwise Tanimoto coefficient (Tc), calculated using the 1,024-bit version of Daylight like 2D-fingerprints with the Open Babel package [51]. For two ligands A and B with fingerprints *f_A_* and *f_B_*, Tc = *f_A_*∩*f_B_*/*f_A_*∪*f_B_*, where symbols ∩ and ∪ represent intersection and union of non-zero bits, respectively.

### Pocket comparison

Structural comparison of pockets was conducted using the program APoc described previously [28]. Here, we give a brief description of the main ideas. Given two input pockets, a template and a target, APoc evaluates their Pocket Similarity score (PS-score), which measures the similarity in their backbone geometries, side-chain orientations, and the chemical similarities between the aligned pocket-lining residues. The length of a pocket is the number of Cα atoms of the pocket residues. Suppose an alignment is obtained between a query (target) of length *L_Q_* and a template of length *L_T_*. The PS-score of the alignment defined as
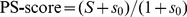
(1)

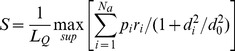
(2)

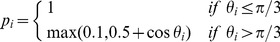
(3)

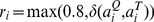
(4)where *N_a_* is the number of aligned residue pairs, *d_i_* is the distance in Å between the Cα atoms of the *i*th aligned residue pair, and the empirical scaling factor 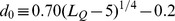. The constants in *d*_0_ were obtained by fitting the distribution of Cα distances in random alignments of pockets. *p_i_* measures the directional similarity between two Cα to Cβ vectors in the two pockets, which span an angle *θ_i_* at the *i*th alignment position of two non-Glycine residues. For Glycine, the value of *p_i_* is assigned 1 if both amino acids are Glycine and 0.77 if only one residue is Glycine. The latter is the mean *p_i_* derived from random alignments. *r_i_* measures the chemical similarity of the two aligned amino acids. 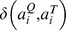 has a value of 1 if the two amino acids 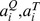 belong to the same group (I–VIII) defined as: I (LVIMC), II (AG), III (ST), IV (P), V (FYW), VI (EDNQ), VII (KR), VIII (H) [52], and 0 otherwise. The scaling factor 
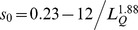
 ensures that the mean score of two aligned random pockets is independent of their length. To calculate the distances used in *d_i_* and *p_i_*, aligned residues are superimposed using the Kabsch algorithm [53] to minimize the RMSD of the full or subset of aligned residues. In principle, the number of all possible superpositions exponentially increases as the alignment length grows. The notation “max” in Eq. 2 indicates that the PS-score corresponds to the superposition that gives the maximum of all scores. In practice, a heuristic iterative extension algorithm is employed to calculate the PS-score, similar to that used for calculating the TM-score [33]. Note that identical pocket structures have a PS-score of 1.0, which is the upper bound of the PS-score.

APoc optimizes the pocket structural alignment through three phases: In the first phase, several guessed solutions are generated from gapless alignments, secondary structure comparisons, fragment alignments, and local contact pattern alignments. Starting from these guessed “seed” alignments, dynamic programming is iteratively applied in the second phase. This yields an “optimal” sequential (*viz*. protein sequence order dependent) alignments between two pocket structures. In the third phase, an iterative procedure searches for the best non-sequential alignment between two pockets, which is then selected if this alignment has a better PS-score than the “optimal” sequential alignment. The problem of finding an optimal non-sequential alignment (or match) is converted to the Linear Sum Assignment Problem (LSAP), which is a special case of integer programming and is also equivalent to the problem of finding a maximum weight matching in a weighted bipartite graph. To efficiently solve LSAP, we implemented the shortest augmenting path algorithm [54], which has a polynomial time complexity of O(*N* ^3^), where 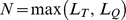.

Since the PS-score is an optimal score from many alignment trials, its distribution can be modeled by the type I extreme value distribution (Gumbel distribution). Using this statistical model, the statistical significance, i.e. *P*-values, of the PS-score is estimated. Parameters of the statistical models were obtained through comparing millions of randomly selected pocket pairs [28].

### Graph analysis

Given a graph *G*, the domination number *N* is defined as the cardinality of the smallest dominating set of the graph. Since the calculation of *N* is a NP hard problem, we implemented a greedy algorithm to estimate this value as follows [55]: For a given set of nodes, the node with the largest number of matched nodes is selected first (two nodes are considered matched if they are connected in both directions in a directed graph). Then, after removing the selected node, the node in the remaining set with the highest number of matched nodes among unmatched nodes is selected. The process is iterated until all nodes that can be matched to the selected set of nodes are identified. The resulting number of this selected set is *N*.

A strongly connected graph is a subgraph where all nodes are bidirectionally connected. The size of the LSCC was calculated using the igraph package for the statistical platform R. The fraction of matching pockets is the ratio of the number of pockets assigned to the dominating set divided by the total number of pockets.

### Protein-ligand interactions

The classification of atomic ligand-protein interactions is obtained from the LPC [50]. For each atomic contact, the associated contact surface area is used to calculate the fraction of conserved contacts. The overall contribution of each type of interaction is calculated as the total contact surface area of each type divided by the total contact surface area for all pockets. When comparing two pairs of protein-ligand interactions, the fraction of conserved interactions for interaction type *i* is defined as 
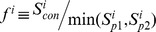
, where 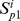 and 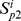 are the total contact surface areas for pocket *p*1 and *p*2, respectively, and 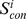 is the contact surface area of conserved contacts.

### Availability

The data set is available at http://cssb.biology.gatech.edu/pocketlib.

## Supporting Information

Figure S1 Statistics of pocket comparisons between representative templates and their matched targets. Cumulative fraction of pocket pairs up to various (**A**) PS-score *P*-value, (**B**) alignment RMSD, and (**C**) alignment coverage, given by the length of alignment divided by the length of the target.
(TIF)

Table S1 **Significance of the PS-score for protein pockets of various lengths.**
(DOCX)

Table S2 **Statistics of representative pockets for most frequent ligands in the PDB.**
(DOC)
