# Supplementary figures and images for: A Comprehensive Survey of Small-Molecule Binding Pockets in Proteins
Source: PLoS Comput Biol. 2013 Oct 24;9(10):e1003302. doi: 10.1371/journal.pcbi.1003302 (PMC3812058; doi:10.1371/journal.pcbi.1003302)

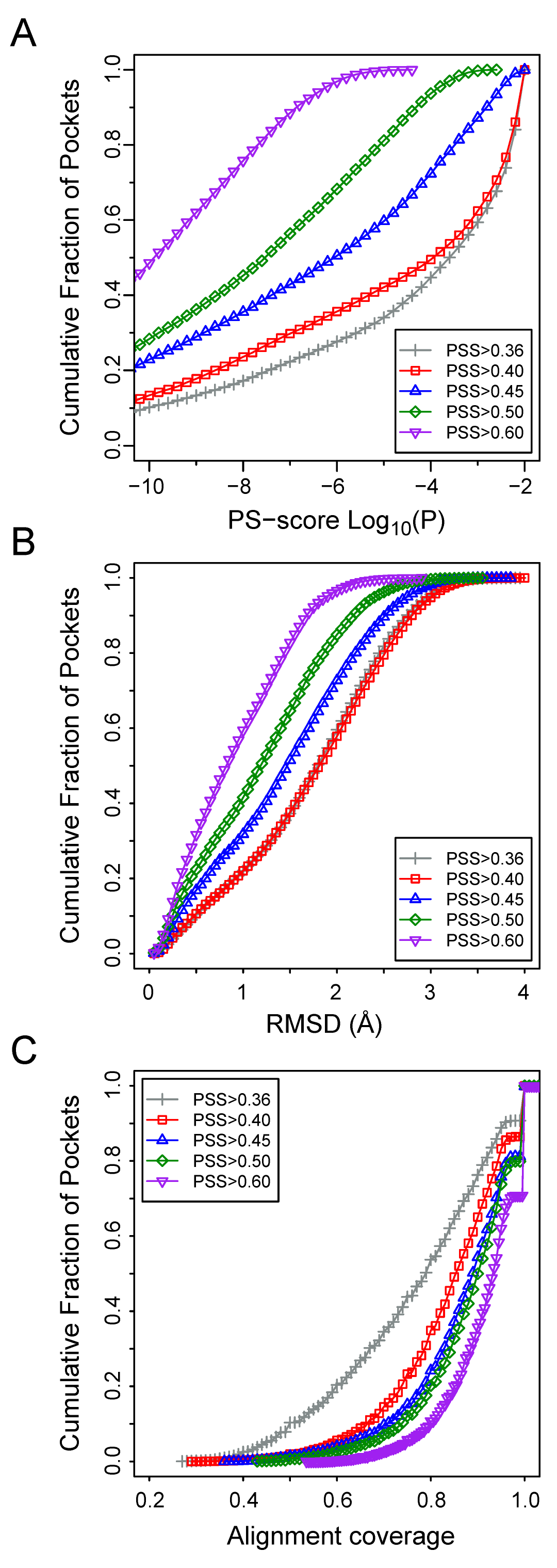

Supplement: Figure S1 — Statistics of pocket comparisons between representative templates and their matched targets. Cumulative fraction of pocket pairs up to various (A) PS-score P-value, (B) alignment RMSD, and (C) alignment coverage, given by the length of alignment divided by the length of the target. (TIF) [file pcbi.1003302.s001.tif]
