# Supplementary material for: A Comprehensive Survey of Small-Molecule Binding Pockets in Proteins
Source: PLoS Comput Biol. 2013 Oct 24;9(10):e1003302. doi: 10.1371/journal.pcbi.1003302 (PMC3812058; doi:10.1371/journal.pcbi.1003302)
Supplement: Table S1 — Significance of the PS-score for protein pockets of various lengths. (DOCX) [file pcbi.1003302.s002.docx]

**Table S1.** Significance of the PS-score for protein pockets of various lengths.

| PS-score | *P*-value | | | | |
| --- | --- | --- | --- | --- | --- |
|  | **15 AAs** | **25 AAs** | **35 AAs** | **45 AAs** | **55 AAs** |
| 0.35 | 1.23×10^-1^ | 8.57×10^-2^ | 6.21×10^-2^ | 4.59×10^-2^ | 3.42×10^-2^ |
| 0.36 | 7.78×10^-2^ | 4.95×10^-2^ | 3.32×10^-2^ | 2.29×10^-2^ | 1.60×10^-2^ |
| 0.38 | 3.03×10^-2^ | 1.62×10^-2^ | 9.35×10^-3^ | 5.61×10^-3^ | 3.43×10^-3^ |
| 0.40 | 1.16×10^-2^ | 5.22×10^-3^ | 2.61×10^-3^ | 1.37×10^-3^ | 7.34×10^-4^ |
| 0.45 | 1.03×10^-3^ | 3.06×10^-4^ | 1.06×10^-4^ | 3.99×10^-5^ | 1.55×10^-5^ |
| 0.50 | 9.13×10^-5^ | 1.79×10^-5^ | 4.34×10^-6^ | 1.16×10^-6^ | 3.26×10^-7^ |
| 0.55 | 8.08×10^-6^ | 1.05×10^-6^ | 1.77×10^-7^ | 3.38×10^-8^ | 6.88×10^-9^ |
| 0.60 | 7.15×10^-7^ | 6.11×10^-8^ | 7.20×10^-9^ | 9.85×10^-10^ | 1.45×10^-10^ |
