# Supplementary material for: A Comprehensive Survey of Small-Molecule Binding Pockets in Proteins
Source: PLoS Comput Biol. 2013 Oct 24;9(10):e1003302. doi: 10.1371/journal.pcbi.1003302 (PMC3812058; doi:10.1371/journal.pcbi.1003302)
Supplement: Table S2 — Statistics of representative pockets for most frequent ligands in the PDB. (DOC) [file pcbi.1003302.s003.doc]

Table S2. Statistics of representative pockets for most frequent ligands in the PDB.

| **Code** | **N** | **0.05** | **0.01** | **1E-03** | **1E-04** | **Name** |  |
| --- | --- | --- | --- | --- | --- | --- | --- |
| **ADP** | 456 | 31 | 56 | 73 | 90 | Adenosine-5'-diphosphate | |
| **HEM** | 438 | 19 | 39 | 57 | 68 | Protoporphyrin IX containing Fe | |
| **NAD** | 371 | 23 | 28 | 33 | 37 | Nicotinamide-adenine-dinucleotide | |
| **FAD** | 329 | 15 | 20 | 22 | 25 | Flavin-adenine dinucleotide | |
| **ATP** | 281 | 40 | 60 | 68 | 82 | Adenosine-5'-triphosphate | |
| **NAP** | 232 | 15 | 19 | 22 | 28 | NADP nicotinamide-adenine-dinucleotide phosphate | |
| **AMP** | 201 | 42 | 62 | 72 | 84 | Adenosine monophosphate | |
| **ANP** | 197 | 23 | 29 | 36 | 51 | Phosphoaminophosphonic acid-adenylate ester | |
| **GDP** | 197 | 16 | 21 | 23 | 24 | Guanosine-5'-diphosphate | |
| **FMN** | 195 | 15 | 22 | 27 | 33 | Flavin mononucleotide | |
| **PLP** | 188 | 9 | 11 | 14 | 16 | Pyridoxal-5'-phosphate | |
| **SAH** | 172 | 8 | 8 | 10 | 14 | S-adenosyl-l-homocysteine | |
| **NAG** | 165 | 49 | 98 | 118 | 124 | N-acetyl-d-glucosamine | |
| **SAM** | 145 | 13 | 13 | 15 | 20 | S-adenosylmethionine | |
| **NDP** | 145 | 13 | 18 | 21 | 27 | NADPH dihydro-nicotinamide-adenine-dinucleotide phosphate | |
| **COA** | 142 | 29 | 40 | 46 | 51 | Coenzyme A | |
| **CIT** | 142 | 52 | 95 | 115 | 124 | Citric acid | |
| **PG4** | 141 | 53 | 98 | 136 | 140 | Tetraethylene glycol | |
| **EPE** | 115 | 72 | 101 | 106 | 108 | 4-(2-hydroxyethyl)-1-piperazine ethanesulfonic acid | |
| **MES** | 110 | 50 | 95 | 102 | 104 | 2-(n-morpholino)-ethanesulfonic acid | |
| **GSH** | 93 | 17 | 18 | 20 | 24 | Glutathione | |
| **PGE** | 83 | 45 | 71 | 79 | 83 | Triethylene glycol | |
| **GNP** | 81 | 5 | 6 | 6 | 7 | Phosphoaminophosphonic acid-guanylate ester | |
| **BGC** | 79 | 40 | 50 | 52 | 55 | Beta-d-glucose | |
| **UDP** | 75 | 17 | 25 | 29 | 41 | Uridine-5'-diphosphate | |
| **GTP** | 72 | 21 | 23 | 26 | 27 | Guanosine-5'-triphosphate | |
| **ACO** | 71 | 16 | 18 | 20 | 20 | Acetyl coenzyme *A | |
| **GLC** | 65 | 27 | 37 | 39 | 44 | Alpha-d-glucose | |
| **1PE** | 64 | 31 | 55 | 61 | 63 | Pentaethylene glycol | |
| **HEC** | 63 | 5 | 13 | 17 | 23 | Heme C |  |
| **PLM** | 59 | 16 | 28 | 43 | 46 | Palmitic acid | |
| **ADN** | 58 | 18 | 21 | 22 | 25 | Adenosine | |
| **GLU** | 51 | 21 | 25 | 27 | 29 | Glutamic acid | |
| **FLC** | 51 | 32 | 45 | 49 | 49 | Citrate anion | |
| **BOG** | 49 | 17 | 28 | 38 | 45 | B-octylglucoside | |
| **TLA** | 48 | 27 | 38 | 41 | 44 | L(+)-tartaric acid | |
| **5GP** | 48 | 13 | 18 | 20 | 20 | Guanosine-5'-monophosphate | |
| **ACP** | 45 | 9 | 14 | 16 | 21 | Phosphomethylphosphonic acid adenylate ester | |
| **P6G** | 45 | 27 | 36 | 45 | 45 | Hexaethylene glycol | |
| **AKG** | 43 | 11 | 11 | 12 | 13 | 2-oxoglutaric acid | |
| **NAI** | 42 | 5 | 6 | 7 | 8 | 1,4-dihydronicotinamide adenine dinucleotide | |
| **SUC** | 38 | 22 | 30 | 30 | 30 | Sucrose |  |
| **MAL** | 37 | 16 | 19 | 20 | 20 | Maltose |  |
| **GAL** | 37 | 20 | 24 | 27 | 27 | Beta-d-galactose | |
| **ARG** | 36 | 19 | 21 | 21 | 23 | Arginine |  |
| **ADE** | 35 | 15 | 18 | 19 | 19 | Adenine |  |
| **U5P** | 34 | 17 | 19 | 22 | 22 | Uridine-5'-monophosphate | |
| **PMP** | 34 | 3 | 3 | 4 | 7 | 4'-deoxy-4'-aminopyridoxal-5'-phosphate | |
| **TPP** | 31 | 5 | 4 | 6 | 6 | Thiamine diphosphate | |
| **TRP** | 31 | 12 | 15 | 16 | 17 | Tryptophan | |
| **STU** | 31 | 1 | 1 | 1 | 1 | Staurosporine | |

**Note:** Code is three-letter ID defined in the PDB for each ligand; N is the total number of pockets for each type of ligand; for each ligand, the number of representative pockets is provided at PS-score *P-values* of 0.05, 0.01, 0.001, and 0.0001, respectively.
